# Patient Safety in Surgery Reviewer acknowledgement 2014

**DOI:** 10.1186/s13037-015-0059-4

**Published:** 2015-03-21

**Authors:** Philip F Stahel, Pierre-Alain Clavien

**Affiliations:** Denver Health Medical Center, Denver, USA; University Hospital Zurich, Zurich, Switerzland

## Abstract

The Editors of Patient Safety in Surgery would like to thank all our reviewers who have contributed to the journal in Volume 8 (2014).

**Negar Ahmadi**

Canada

**Emma-Louise Aveling**

United Kingdom

**Fernando Baldy**

Brazil

**Rodrigo Banegas**

United States of America

**William Berry**

United States of America

**Shay Bess**

United States of America

**Walter L. Biffl**

United States of America

**Frank Bode**

Germany

**Nathan Butler**

United States of America

**Mark Chandler**

United States of America

**Qi Chen**

United States of America

**Ted Clarke**

United States of America

**Muhammad Muzzammil Edhi**

Pakistan

**Vincent Eusterman**

United States of America

**Johannes Fakler**

Germany

**Joan Figueras**

Spain

**Ingo Fiss**

Germany

**Michael Flierl**

United States of America

**Charles Fox**

United States of America

**Beat Gloor**

Switzerland

**Dieter Hahnloser**

Switzerland

**Eric Mark Hammerberg**

United States of America

**Jiandong Hao**

United States of America

**Markus Huber-Lang**

Germany

**Kyros Ipaktchi**

United States of America

**Ratna Johari**

India

**Jeffrey Johnson**

United States of America

**Jeffry Kashuk**

United States of America

**Sebastian Katscher**

Germany

**Fernando Kim**

United States of America

**Kodi Kojima**

Brazil

**Christian K. Lackner**

Germany

**Susan Ladley**

United States of America

**Philipp Lenzlinger**

Switzerland

**Shalin Maheshwari**

India

**Matthias Majetschak**

United States of America

**Cyril Mauffrey**

United States of America

**Howard Miller**

United States of America

**Wilson Molina**

United States of America

**Philipp Mommsen**

Germany

**John Mukhopadhaya**

India

**Kenneth Nepple**

United States of America

**Justin Newman**

United States of America

**Giuseppe Nigri**

Italy

**Lena Nilsson**

Sweden

**Andrew Robson**

United Kingdom

**James Ruskin**

United States of America

**Stefan Sauerland**

Germany

**Samuel Smith**

United States of America

**Wade Smith**

United States of America

**Olaf Suess**

Germany

**Peter Teschendorf**

Germany

**Per Trobisch**

Germany

**Todd VanderHeiden**

United States of America

**Michael Victoroff**

United States of America

**Robert Waltz**

United States of America

**Sebastian Weckbach**

United States of America

**Allison Williams**

United States of America

**Heather Young**

United States of America

**Bruce Ziran**

United States of America

